# miR-22 inhibits mouse ovarian granulosa cell apoptosis by targeting SIRT1

**DOI:** 10.1242/bio.016907

**Published:** 2016-02-24

**Authors:** Fang Xiong, Lingqing Hu, Yun Zhang, Xiao Xiao, Juxia Xiao

**Affiliations:** Procreation and Medicine Centre, Wuxi Maternal and Child Health Hospital, Wuxi 214002, China

**Keywords:** SIRT1, miR-22, Granulosa cell, Apoptosis, Follicle atresia

## Abstract

Granulosa cell (GC) apoptosis has been shown to be involved in follicular atresia, which is a degenerative process in ovarian follicles of mammals. However, the mechanism underlying the regulation of follicular atresia, particularly by microRNAs, is not well known. Real-time PCR (RT-PCR) was used to detect the expression level of miR-22 in healthy follicles (HF), early atretic follicles (EAF), and progressively atretic follicles (PAF). Flow cytometry was performed to assess the apoptosis of mouse granulosa cells (mGCs) treated with miR-22 mimics or negative control (NC) mimics. Regulation of the expression of SIRT1 by miR-22 was evaluated using a luciferase reporter assay system. To investigate the roles of SIRT1 in mGC apoptosis, the endogenous SIRT1 gene in mGCs was knocked down using an siRNA specific for SIRT1. miR-22 was increased during follicular atresia and suppressed granulosa cell apoptosis. The results of the luciferase reporter assay indicated that SIRT1 was a target gene of miR-22. In addition, knockdown of SIRT1 attenuated apoptosis in mGCs. miR-22 inhibits mGC apoptosis by downregulating SIRT1 directly *in vitro*. This study provides important insights into understanding the regulation mechanism of ovarian follicle atresia.

## Abstract (precis)

**Summary:** Overexpression of miR-22 inhibits mGC apoptosis by targeting SIRT1, and knockdown of SIRT1 attenuated apoptosis in mGCs. Taken together, these findings provide an improved understanding of the molecular mechanisms of miR-22-mediated follicular development.

## INTRODUCTION

A large number of follicles are present in the mammalian ovary at birth, and each follicle contains an oocyte that is surrounded by several layers of granulosa cells (GCs). Follicular atresia is a complicated retrogressive process at various stages of development and is controlled by endocrine and paracrine factors. Numerous evidence has demonstrated that GC apoptosis is a main mechanism for follicular atresia (Manabe et al., 2004). A high incidence of GC apoptosis has been demonstrated to result in increased empty follicles, fewer oocyte retrieval, and decline of oocyte quality (Sun et al., 2012). Hormones, cytokines, and growth factor play an important role in process of GC apoptosis during follicular atresia (Kosaka et al., 2007; Matsuda-Minehata et al., 2006; Wang et al., 2012). However, the mechanism of genetic regulation on follicular atresia is not well known.

Silent mating-type information regulation 2 homologue 1 (SIRT1), a member of class III histone deacetylase family, plays a regulatory role in cell proliferation, differentiation, apoptosis, metabolic homeostasis, and oxidative stress in multiple cell types (Qu et al., 2012; Srisuttee et al., 2012; Xia et al., 2012). SIRT1 protein has been detected to express in human ovarian tissues and nuclei of human luteinized GCs at different stages of follicular development (Morita et al., 2012). A recent study suggested that over-expression of SIRT1 promoted to chemoresistance and was associated with a poor prognosis in serous epithelial ovarian cancer (Shuang et al., 2015). SIRT1 exhibits the anti-apoptosis effect by reducing expression of pro-apoptotic proteins Bax, p53 and stimulating expression of anti-apoptotic protein Bcl-xL (Cohen et al., 2004; Seo et al., 2012). On the other hand, SIRT1 has also been shown to have a pro-apoptotic capability. The apoptotic rate was significantly increased in GCs treated with resveratrol, which was an activator of SIRT1. Meanwhile, the mRNA levels of pro-apoptosis markers including caspase-3 and Bax were significantly elevated and the mRNA level of Bcl-2, a anti-apoptosis marker, was significantly decreased in GCs (Zhao et al., 2014). Moreover, activation of SIRT1 by resveratrol inhibited rat GC viability in a concentration-dependent manner (Morita et al., 2012).

MicroRNAs (miRNAs) are a class of short non-coding endogenous RNAs of approximately 22 nucleotides in length and play important roles in multiple biological and cellular processes such as cell growth, differentiation, apoptosis, development, and tumorigenesis (Cimmino et al., 2005; Lim et al., 2005; Yang et al., 2012a). miRNAs posttranscriptionally regulate the expression of most protein-coding genes by binding to the 3′-untranslated regions of target mRNAs, resulting in either mRNA degradation or sequence-specific translational repression. The decreased miR-92a expression level of was observed in atretic porcine follicles and transfection of GCs with miR-92a mimics significantly attenuated porcine GC apoptosis by targeting Smad7 gene (Liu et al., 2014). A previous study suggested that miR-22 was capable of inhibiting neuronal apoptosis, as shown by its capability to attenuate activation of effector caspases (Jovicic et al., 2013). The expression level of Bax, a apoptosis marker, were significantly decreased in human ovarian GCs transfected with miR-22, suggesting that miR-22 may inhibit ovarian GC apoptosis (Sirotkin et al., 2010). Identification of putative target genes of ovarian miRNAs will contribute to our knowledge of miRNAs in the regulation of folliculogenesis.

In the present study, we investigated the expression of miR-22 in mouse ovarian GCs during follicular atresia. We further evaluated the effect of miR-22 on SIRT1 expression and elucidated the functional role of SIRT1 in apoptosis regulation.

## RESULTS

### miR-22 expression level was decreased during follicular atresia in mice

**Fig. 1.**
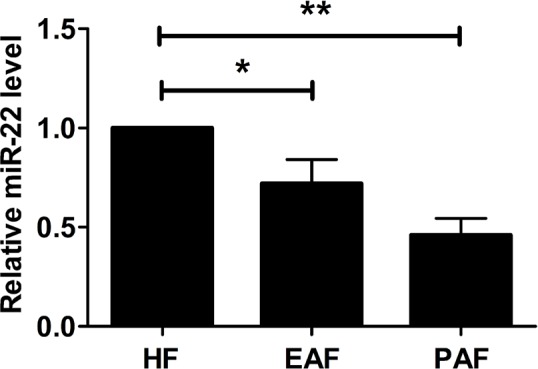
**The expression of miR-22 in mouse ovarian granulosa cells.** The relative expression levels of miR-22 were measured in healthy follicles (HF), early atretic follicles (EAF), and progressively atretic follicles (PAF) using qRT-PCR. U6 was used as a loading control to normalize expression levels. Data were expressed as the mean±s.d. (*n*=3). *P<0.05, **P<0.01.

RT-PCR was conducted to detect the expression levels of mature miR-22 in healthy follicles (HF), early atretic follicles (EAF), and progressively atretic follicles (PAF). The data showed that miR-22 was significantly decreased during mouse follicular atresia (Fig. 1). These results demonstrated that miR-22 may be involved in apoptosis in mouse ovarian follicles.

### miR-22 inhibits GC apoptosis

**Fig. 2.**
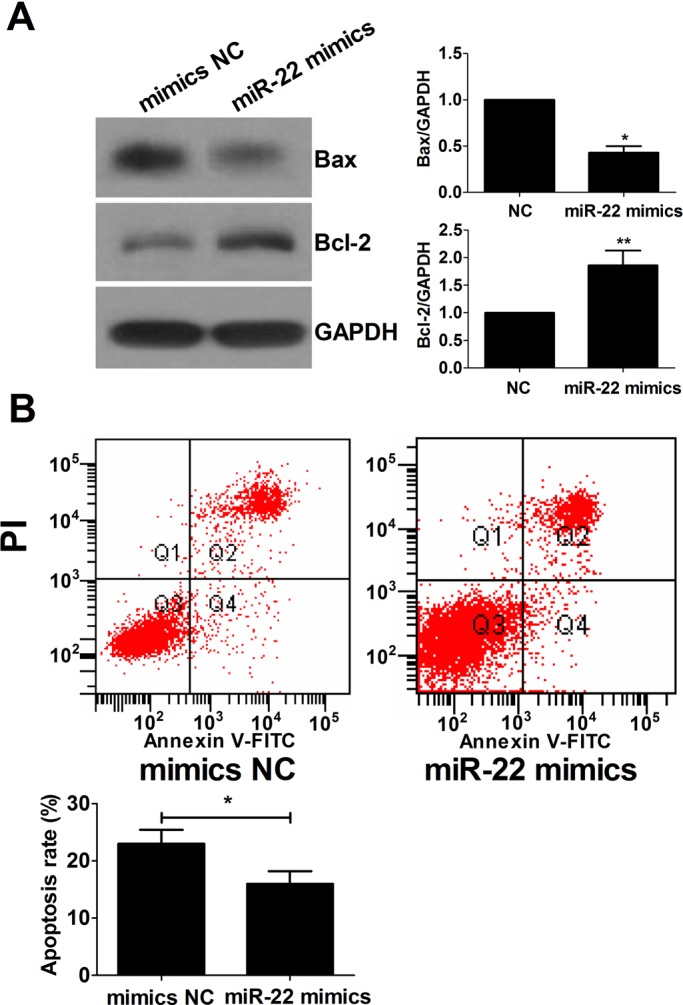
**Apoptosis rate of mGCs when transfecting with miR-22 mimics or mimics NC.** (A) Western blot was performed to detect the expression levels of Bax and Bcl-2. (B) mGC apoptosis level after miR-22 mimics or mimics NC treatment was determined by FACS. Data were expressed as the mean±s.d. (*n*=3). *P<0.05, **P<0.01.

GCs play a major role in maintaining the balance between follicular growth and apoptosis in mammalian ovaries (Matsuda et al., 2012). To investigate the potential function of miR-22 in GC apoptosis in the mouse ovary, the isolated mGCs were incubated *in vitro* and transiently transfected with miR-22 mimics. Decreased Bax expression and increased Bcl-2 expression were observed in the miR-22 mimics group (Fig. 2A). The apoptosis levels were assessed using Annexin V-FITC/PI double staining method. As shown in Fig. 2B, the apoptosis rate of mGCs transfected with miR-22 mimics remarkably decreased compared with control groups, suggesting that miR-22 suppressed mGCs apoptosis *in vitro*.

### miR-22 directly inhibits the expression of SIRT1 via binding to its 3′-UTR

**Fig. 3.**
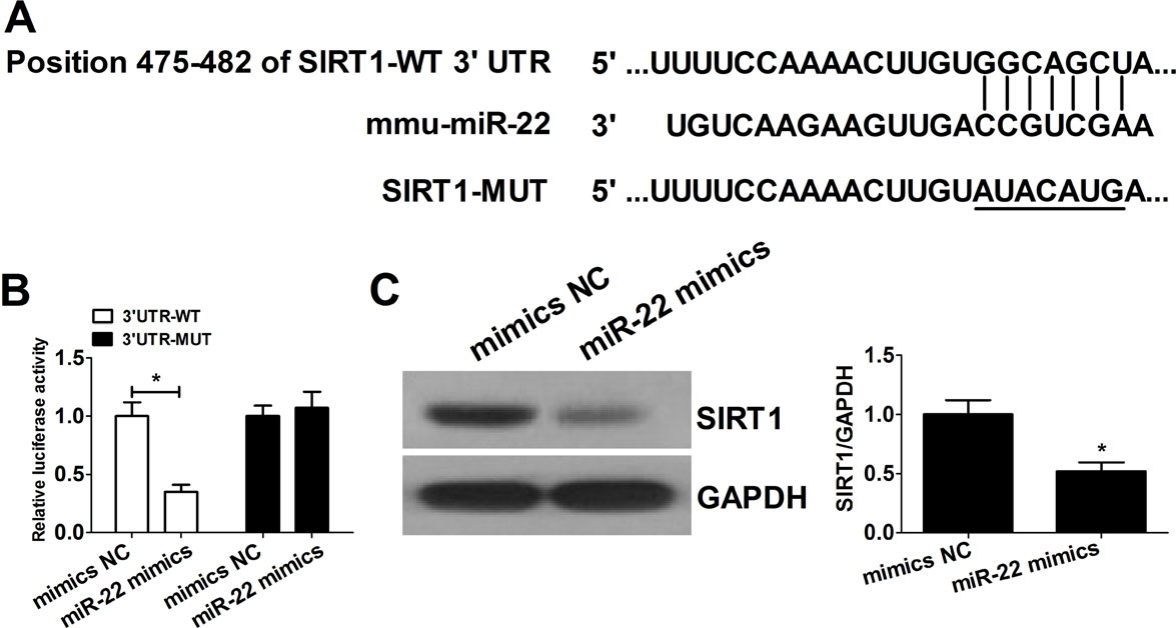
**miR-22 directly inhibits expression of SIRT1 via its 3′-UTR.** (A) Putative binding sites for mouse miR-22 on SIRT1 3′-UTR are shown. The mutated region in SIRT1 3′-UTR is indicated with a horizontal line. (B) HEK293T cells were co-transfected with wild type (WT) or mutant (MUT) SIRT1 3′-UTR luciferase reporter plasmid, along with miR-22 mimics or mimics NC. The relative luciferase activities were detected 24 h after transfection. (C) The protein expression level of SIRT1 was determined by western blot. Data were expressed as the mean±s.d. (*n*=3). *P<0.05.

To confirm SIRT1 was directly targeted and regulated by miR-22 in HEK293 cells, luciferase reporter genes with SIRT1 3′-UTR and the mutant counterpart at the miR-22 binding regions were co-transfected with miR-22 mimics or mimics NC into HEK293 cells. The putative miR-22-binding sites in mouse SIRT1 3′-UTR and the mutant SIRT1 gene 3′-UTR with modified binding sequence were shown in Fig. 3A. Luciferase reporter assay showed that overexpression of miR-22 significantly inhibited the luciferase activity of SIRT1 with the wild-type 3′-UTR, but not with the mutant 3′-UTR (Fig. 3B). As shown in Fig. 3C, the expression of SIRT1 significantly decreased in miR-22 mimics group. These findings demonstrated that miR-22 targeted sites of 3′-UTR region of SIRT1 and that there is an inverse correlation between the miR-22 expression level and the protein expression level of SIRT1. Therefore, SIRT1 was a target gene of miR-22 and SIRT1 may play an important role in the process of miR-22 affecting GC apoptosis.

### Downregulation of SIRT1 suppresses mouse ovarian GC apoptosis

**Fig. 4.**
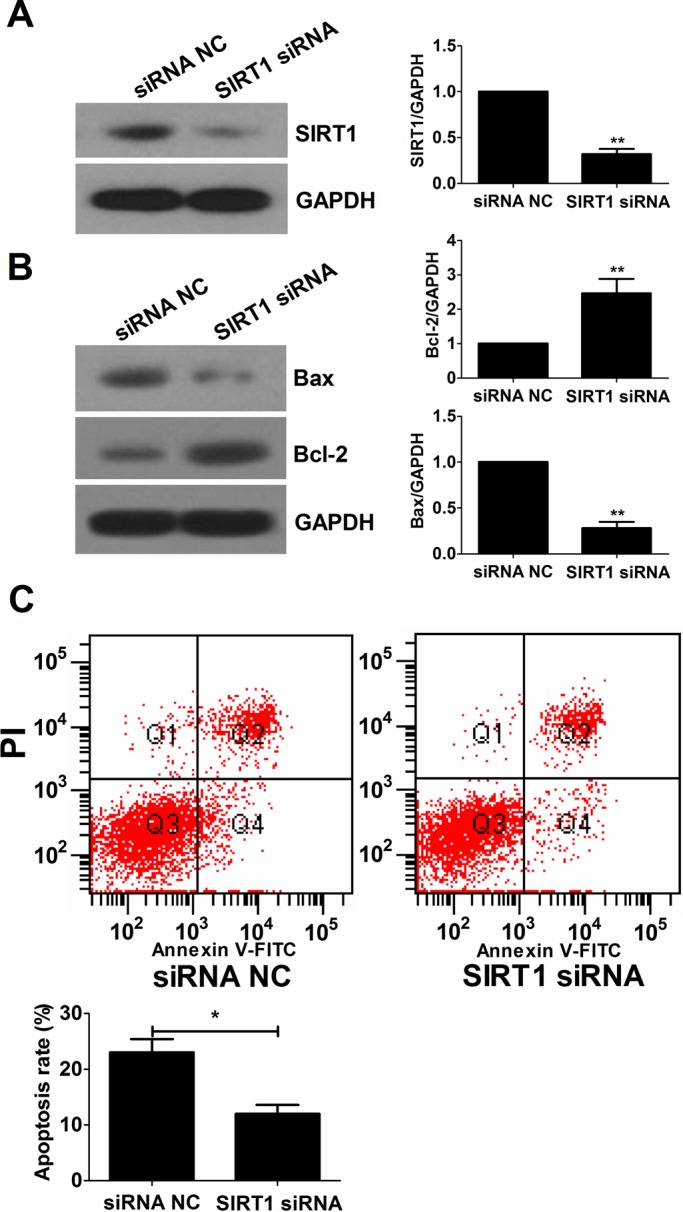
**The effect of SIRT1 knockdown on mGC apoptosis.** (A,B) Western blot was performed to detect the expression levels of SIRT1 (A), Bax and Bcl-2 (B) in mGCs transfected with SIRT1 siRNA or control siRNA. (C) mGC apoptosis level was measured by FACS 48 h after transfection with SIRT1 siRNA or control siRNA. Data were represented as the mean±s.d. (*n*=3). *P<0.05, **P<0.01.

To examine whether SIRT1 downregulation affects GC apoptosis, endogenous SIRT1 gene in mGCs was knocked down by RNA interference. As shown in Fig. 4A, The expression level of SIRT1 protein was significantly decreased in the SIRT1 siRNA group compared with that in the siRNA NC group. Furthermore, the expression levels of Bax and Bcl-2 were reversed in the SIRT1 siRNA group (Fig. 4B). Knockdown of SIRT1 reduced the apoptosis rate of the GCs (Fig. 4C). These results suggested that SIRT1 may be involved in regulation of follicular atresia by promoting mGC apoptosis.

## DISCUSSION

In the present study, we investigated effects of the miR-22/SIRT1 signaling on the regulation of GC apoptosis*.* The expression level of miR-22 was increased in EAF and PAF. Flow cytometric analysis was performed to evaluate the *in vivo* effect of miR-22 on apoptosis of mouse ovarian GCs. Furthermore, we knocked down the expression of SIRT1 in mGCs. The results showed that downregulation of SIRT1 inhibited apoptosis in mouse granulose cells, suggesting that miR-22 may regulate apoptosis at least partly via the regulation of SIRT1. To our knowledge, we for the first time demonstrated that miR-22 regulated apoptosis through suppressing SIRT1 expression in GC apoptosis.

SIRT1 has been reported to be expressed in ovarian follicular cells (Morita et al., 2012). SIRT1 regulates multiple transcription factors, such as p53, RelA/p65, and the forkhead transcription factor family of proteins by deacetylating lysine residues at the N-terminal extensions of core histones (Brunet et al., 2004; Kim et al., 2007). Pavlová et al. suggested that SIRT1 was able to inhibit NF-κB and served as a mediator of FSH activity in proliferation and secretory activity of ovarian cells (Pavlová et al., 2013). SIRT1 inhibits breast cancer progression and accelerates the apoptosis of human breast cancer cell lines by downregulating the expression of pro-survival Bcl-2 protein (Kuo et al., 2013). SIRT1 has been implicated in apoptosis of a variety of cell types, but the exact role of SIRT1 in follicular development of mammals, especially in GC apoptosis during follicular atresia, still remains rarely known. In this study, we found that SIRT1 was a target gene of miR-22 and inhibition of SIRT1 attenuated mouse ovarian GC apoptosis.

miRNAs have been demonstrated to mediate post-transcriptional regulation of gene expression by binding the specific site of 3′-UTR of genes from plants to mammals (Tüfekci et al., 2014). Transfection with miR-23a downregulated the expression levels of XIAP (X-linked inhibitor of apoptosis protein) and caspase-3 protein and upregulated caspase-3 protein expression in human ovarian GCs, along with an increased apoptosis rate. Some miRNAs abnormally expressed in the serum of premature ovarian failure patients may be implicated in regulation of GC proliferation and apoptosis through various signaling pathways (Yang et al., 2012b). By expression profiling analyses, Ro et al. identified 122 miRNAs expressed in the ovary and 15 novel miRNAs were abundantly expressed in the ovary (Ro et al., 2007). These findings indicated that miRNAs may play crucial roles in the regulation of folliculogenesis and improvement of female fertility. Overexpression of miR-125b-1 significantly promoted human glioma cell viability and inhibited the early and late cell apoptosis after all-trans-retinoic acid treatment (Xia et al., 2009). The expression level of miR-22 was decreased in ovarian cancer tissues compared with healthy human ovarian surface epithelium (Wyman et al., 2009). Furthermore, an experiment in cultured human ovarian GCs showed that transfection with miR-22 precursors remarkably affected expression of proliferation marker, PCNA, and proapoptotic marker, Bax (Sirotkin et al., 2010). To make clear the precise mechanism by which miR-22 attenuated granulose cell apoptosis, we detected the expression level of SIRT1 in mGCs transfected with miR-22 mimics or miR-22 inhibitor. We found that miR-22 down-regulate SIRT1 expression through post-transcriptional regulation via a miR-22-binding site within the 3′-UTR of SIRT1.

In conclusion, our data suggest involvement of SIRT1 in the process of follicle atresia *in vitro*. Overexpression of miR-22 suppressed mouse ovarian GC apoptosis at least in part by blocking SIRT1 expression. With identification of SIRT1 as a target for miR-22 in ovarian GCs, this study provided an improved understanding of molecular mechanisms of miR-22-mediated follicular development, which may potentially be applied in future clinical applications.

## MATERIALS AND METHODS

### Quantitative real-time polymerase chain reaction (qRT-PCR)

Total RNA were extracted from ovarian GCs of mice using a TRIZOL regent kit (Invitrogen, Carlsbad, CA, USA) according to the manufacturer's instructions. The purity of RNA was assessed by a NanoDrop 2000 UV-Vis spectrophotometer (NanoDrop Technologies, Wilmington, DE, USA) at 260/280. Total RNA was reversely transcribed using TaqMan miRNA reverse transcription kit (Applied Biosystems Inc., Foster City, CA, USA) to detect the expression level of miR-22. U6 small nuclear RNA was used as an internal control for miR-22 mRNA expression. The data obtained were calculated by 2^−ΔΔCt^ method and normalized against the control gene. Each experiment was repeated three times.

### Cell culture

HEK293 cells were cultured in Dulbecco's modified Eagle's medium (DMEM, Gibco, Carlsbad, CA, USA) with 10% fetal bovine serum (Gibco), 100 U/ml penicillin (Sigma-Aldrich, Louis, MO, USA), and 100 µg/ml streptomycin (Sigma). The cells were maintained in a cell incubator (Thermo Scientific, Rockford, IL, USA) containing 5% humidified CO_2_ at a temperature of 37°C.

### Isolation and culture of mouse GCs

Ovaries of 21-day-old immature ICR mice were removed and immediately transferred into DMEM/Nutrient Mixture F-12 (Gibco BRL, Rockville, MD, USA) supplemented with 10% fetal bovine serum (Gibco), 100 units/ml penicillin, 100 μg/ml streptomycin and 0.25 μg/ml amphotericin B. mGCs were collected from the ovaries using the follicular puncture method (Kipp et al., 2007). Isolated mGCs were washed twice and centrifuged at 200 ***g*** for 5 min. After purification, mGCs were cultured in DMEM/Nutrient Mixture F-12 medium containing 10% FBS (Gibco), 1% sodium pyruvate (Gibco), 1% glutamine (Gibco), 100 units/ml penicillin, and 100 μg/ml streptomycin at a temperature of 37°C and 5% humidified CO_2_. After 24 h of incubation, cells were washed thoroughly with phosphate-buffered saline (PBS) to remove non-adherent cells. The adherent cells were cultured for the next experiment and the culture medium was replaced every 3 days.

### Flow cytometric analysis of apoptosis

The apoptotic cells were detected by Annexin V-FITC/propidium iodide (PI) double staining method. Cells were washed twice with cold PBS and resuspended in 100 μl of 1× labeling buffer containing PI and FITC-conjugated Annexin V. After incubation for 15 min at room temperature away from light, the stained cells were sorted by fluorescence-activated cell sorting (FACS) using a flow cytometer (BD Biosciences, Franklin Lakes, NJ, USA).

### Transfection and luciferase reporter assay

The luciferase reporter plasmids fused with the 3′-UTR of mouse SIRT1 containing the putative miR-22 binding site were obtained from GenePharma (Shanghai, China). HEK293 cells were seeded in 24-well plates 24 h before transfection. Then the cells were transiently co-transfected with 0.3 µg wild type or mutant SIRT1 3′-UTR luciferase reporter plasmid and 50 nM miR-22 mimics or miR-control using Lipofectamine 2000 transfection reagent (Invitrogen, Carlsbad, CA, USA). At 24 h after transfection, cells were harvested and the luciferase activity of cell lysates was detected using the Luciferase Assay System (Promega, Madison, WI, USA) according to the manufacturer's instructions.

### Western blot analysis

The mGCs were harvested and then lysed using RIPA Lysis buffer (Beyotime Institute of Biotechnology, Jiangsu, China). Proteins mixed with loading buffer were subjected to SDS-PAGE and transferred onto a hybrid polyvinylidene difluoride membrane (PVDF, Millipore Inc., Billerica, MA, USA). Then, non-specific binding was blocked by incubating with 5% (w/v) nonfat milk in Tris-buffered saline (TBS) with Tween-20 (Sigma-Aldrich) at room temperature for 1 h. The transferred membranes were probed with primary antibodies of SIRT1 (Abcam, Cambridge, UK), Bcl-2 (Santa Cruz Biotechnology, Santa Cruz, TX, USA), Bax (Santa Cruz) and GAPDH (Santa Cruz) at 4°C for 12 h. After overnight incubation with the primary antibody, the PVDF membrane was washed with TBS containing 0.05% Tween-20 (TBST) three times (10 min each). Blots were probed with horseradish peroxidase (HRP)-conjugated secondary antibodies (Santa Cruz) for 1 h at room temperature. Following washing three times (15 min each) with TBST at room temperature, the membranes were subjected to enhanced chemiluminescence (Pierce, Rockford, IL, USA) and exposed to film.

### Statistical analysis

Data are presented as the mean±standard deviation (s.d.) of three independent experiments. Student's *t*-test and one-way analysis of variance were used for statistical analyses. The statistical analysis was performed using SPSS 16.0 software (SPSS Inc., Chicago, IL, USA). A *P*-value of less than 0.05 was considered to be statistically significant.
